# The *NEAT1_2*/miR-491 Axis Modulates Papillary Thyroid Cancer Invasion and Metastasis Through TGM2/NFκb/FN1 Signaling

**DOI:** 10.3389/fonc.2021.610547

**Published:** 2021-03-02

**Authors:** Wei Sun, Yuan Qin, Zhihong Wang, Wenwu Dong, Liang He, Ting Zhang, Hao Zhang

**Affiliations:** Department of Thyroid Surgery, The First Hospital of China Medical University, Shenyang, China

**Keywords:** papillary thyroid cancer, lncRNA, TGM2, FN1, NEAT1_2

## Abstract

NEAT1 (nuclear paraspeckle assembly transcript 1) is an oncogenic long non-coding RNA (lncRNA) that facilitates tumorigenesis in multiple cancers. In papillary thyroid cancer (PTC), the molecular mechanism by which *NEAT1* affects invasion and metastasis remains elusive. RNA sequencing was used to discover differentially expressed *NEAT1_2* downstream genes. Protein and RNA expression analyses and immunohistochemistry detected the expression of *NEAT1_2*, Transglutaminase 2 (TGM2), and microRNA-491 (miR-491) among PTC and non-cancerous tissues. Transwell and wound healing assays, and a mouse model of lung metastasis were used for further functional analyses. Bioinformatics was performed to predict miRNAs binding to both *NEAT1_2* and *TGM2*. Rescue experiments and dual-luciferase reporter assays were performed. In PTC tissues, *NEAT1_2* expression was markedly increased and regulated TGM2 expression. TGM2 was overexpressed in PTC, correlating positively with exthyroidal extension and lymph node metastasis. *TGM2* knockdown significantly inhibited invasion and metastasis. *NEAT1_2* sponged miR-491, acting as a competing endogenous RNA to regulate *TGM2* expression. Fibronectin 1 (FN1) was predicted as a TGM2 target. TGM2 could transcriptionally activate FN1 by promoting nuclear factor kappa B (NFκb) p65 nuclear translocation, ultimately promoting PTC invasion/metastasis. These findings identify that *NEAT1_2* sponges miR-491 to regulate TGM2 expression. TGM2 activates FN1 *via* NFκb to promote PTC invasion and metastasis.

## Introduction

Among endocrine malignancies, thyroid cancer is the most common, representing almost 5% of new cancer cases and its incidence has increased rapidly worldwide over the past 20 years (1). Papillary thyroid carcinoma (PTC) accounting for 80 to 90% (2). Despite improvement in detection and surgical management, including surgical resection, radiotherapy, and levothyroxine treatment, among patients with aggressive PTC, 30% will suffer from recurrence and distant metastasis, which can cause death (3, 4). According to American Joint Committee on Cancer 8^th^ stage for differentiated thyroid cancer, local invasion and distant metastasis are critical factors that affect the prognosis of thyroid cancer (5). There are many risk factors that are closely related to PTC initiation and progression, such as genetic factors, environmental exposure, and epigenetic alterations (6). However, its precise mechanism remains elusive. Therefore, understanding the underlying molecular mechanism of PTC invasion and metastasis is essential to identify valuable biomarkers and therapeutic targets for PTC.

Long non-coding RNAs (lncRNAs) comprise RNA transcripts of >200 nucleotides with little or no capacity to encode proteins (7). Recent studies have reported that lncRNAs can function as molecular sponges, scaffolds, and guides in interactions with microRNAs (miRNAs), proteins, and mRNAs, resulting in complex networks that modulate a variety of cancer phenotypes (8). *NEAT1* (nuclear paraspeckle assembly transcript 1) is an lncRNA that was discovered recently to be a vital component of nuclear paraspeckles, and is dysregulated in multiple solid cancers (9–11). Previously, we reported that knockdown of *NEAT1_2* induced apoptosis and inhibited migration by regulating ATPase family AAA domain containing 2 (ATAD2) expression in PTC (12). However, rescue experiments indicated that ATAD2 was not the only downstream target of *NEAT1_2*. This means that there must be other downstream targets that mediate the invasion and metastasis of PTC.

The transglutaminase family of enzymes includes transglutaminase 2 (TGM2), a calcium-dependent cross-linking enzyme that uses transamidation to catalyze protein modifications, which promotes the formation of polyaminated proteins or lysine combinations in the presence of calcium (13, 14). TGM2 functions in many biological processes, such as extracellular matrix stabilization, cell differentiation, maintenance of cancer stem cell survival, and invasive and metastatic behavior (15–19). However, the expression and function of TGM2 in PTC is unknown.

Research has shown that lncRNAs might function as competing endogenous RNAs (ceRNAs) to modulate microRNAs (miRNAs). As an oncogene, *NEAT1* promotes docetaxel resistance in prostate cancer by regulating acyl-CoA synthetase long chain family member 4 (ACSL4) *via* sponging miR-34a-5p and miR-204-5p (9). Moreover, *NEAT1* could contribute to cell proliferation, apoptosis, and invasion in lung cancer *via* the miR-1224/Kruppel like factor 3 (KLF3) axis, and promoted autophagy by regulating miR-204/autophagy related 3 (ATG3) and enhanced cell resistance to sorafenib in hepatocellular carcinoma (10, 11). Taken together, *NEAT1* could play a critical role in human cancers by inhibiting the effects of miRNAs.

In the present study, we initially assessed the regulatory relationship between TGM2 and *NEAT1_2*. We then analyzed TGM2’s function and expression in PTC cell lines and tissues. Further mechanistic research identified that *NEAT1_2* might act as a competing endogenous RNA (ceRNA) that modulates miR-491 levels to regulate *TGM2*. Furthermore, TGM2 could regulate PTC invasion and metastasis by promoting nuclear factor kappa B (NFκb) p65 nuclear translocation, resulting in transcriptional activation fibronectin 1 (FN1). The results revealed that *NEAT1_2* affects PTC progression by multiple mechanisms; thus, our study contributes to the characterization of the detailed molecular mechanisms of invasion and metastasis in PTC.

## Materials and Methods

### Collection of Patient Specimens and PTC Cell Lines

We collected 174 paired PTC tissues samples and their adjacent non-cancerous thyroid tissue samples from patients who attended the First Hospital of China Medical University between 2011 and 2017. Eighty pairs of PTC tissues and adjacent non-cancerous was used in quantitative real-time reverse transcription PCR (qRT-PCR), 92 pairs were used for immunohistochemistry, and 12 pairs was used for western blotting. For qRT-PCR and western blotting, all tissue specimens were confirmed by two pathologists independently, frozen in liquid nitrogen immediately after surgical resection, and then stored at −80°C until later use. The Ethics Committee of the First Affiliated Hospital of China Medical University, Shenyang, China approved this study. Written informed consent was obtained from all study participants. Two PTC verified *NEAT1_2* overexpressing cell lines (compared with that in Nthy-ori 3-1 cells, a normal human thyroid follicular epithelial cell line), K1 and TPC1, were used in this study. The source and culture method of the cell lines were the same as those reported in previous studies (12).

### RNA Extraction, Sequencing, and Expression Quantification

Total RNA was extracted from three pairs of NC or si-NEAT1_2 K1 (K1 cells transfected to express a small interfering RNA targeting *NEAT1_2*) or NC (negative control) cells, and then the mRNA was enriched using Oligo(dT) beads. Further mRNA enrichment was achieved by removing rRNA by Ribo-ZeroTM Magnetic Kit (Epicentre). TopHat2 (version 2.1.1) was used to map the reads from each sample to the reference genome. RSEM software was then used to quantify expression. Transcripts were identified as differentially expressed genes (DEGs) if they had a fold change ≥ 2 and a false discovery rate (FDR) < 0.05.

### Plasmids, Cell Transfection, and Lentivirus Infection

Gene Pharma (Suzhou, China) provided the *NEAT1_2* and NC siRNAs. The sequences were si-NEAT1_2 (sense): 5′-GGA GGA GUC AGG AGG AAU AUU-3′, si-TGM2 (sense): 5′- GCC TCG TGG TTA TTA GCA AGG -3′, and si-p65 (sense): 5′- GGA GTA CCC TGA GGC TAT AAC TCG C -3′. The sequence of the negative control (NC) was 5′-UUC UCC GAA CGU GUC ACG UUU-3′. The miR-135b-5p mimic, miR-491 mimic, and miR-19b-3p mimic were purchased from Gene Pharma. The pcDNA-TGM2 plasmid and the pcDNA empty plasmid were purchased from GeneChem (Shanghai, China). Transfections were performed using Lipofectamine 2000 (Invitrogen, Carlsbad, CA, USA) according to the manufacturer’s instruction. Recombinant lentiviruses containing a *NEAT1_2* small hairpin RNA (shRNA) or the control were purchased from Obio Technology (Shanghai, China). For shRNA lentivirus infection, cells were infected in six-well plates and subsequently split into 75 cm^2^ cell culture bottle in the presence of 2 μg/ml puromycin for selection over 96 h. The stably transfected cell lines were identified using qRT-PCR.

### Total RNA Extraction and qRT-PCR

RNAiso (Takara, Dalian, China) was used to extract total RNA from frozen samples of tissues and cells. PrimeScript^™^ RT Master Mix and SYBR^®^ Premix Ex Taq^™^ II (Takara) in the Light Cycler 480 system (Roche, USA) were used to perform the qRT-PCR reactions. The primers used comprised NEAT1_2 (sense): 5′-CTA GAG GCT CGC ATT GTG TG-3′; NEAT1_2 (antisense): 5′-GCC CAC ACG AAA CCT TAC AT-3′. TGM2 (sense): 5′-ATA AGT TAG CGC CGC TCT CC-3′; TGM2 (antisense): 5′-CCA GCT CCA GAT CAC ACC TC-3′. GAPDH (sense): 5′-CCG GGA AAC TGT GGC GTG ATG G-3′; GAPDH (antisense): 5′-AGG TGG AGG AGT GGG TGT CGC TGT T-3′. miR-491 (sense): 5′-AGU GGG GAA CCC UUC CAU GAG G-3′. The miRNA antisense primer was provided by the 638313 Mir-X™ miRNA First-Strand Synthesis Kit (Takara). The primers for the endogenous control gene U6 were: U6 (sense): 5′-TCG GCA GCA CAT ATA CTA A-3′; U6 (antisense): 5′-AAC ATG GAA CGC TTC ACG AAT-3′. The relative expression levels were calculated using the 2^-ΔCT^ method (CT, cycle threshold).

### Transwell Assay

After transfection of K1 and TPC1 cells for 24 h, the cell concentration in each group was adjusted to 1.5 × 10^4^ cells/ml using serum-free medium. The upper Transwell chamber was filled with 200 μl of cell suspension with or without Matrigel™ (Corning, Corning, NY, USA), and the lower Transwell chamber was filled with 600 μl of medium containing 10% fetal bovine serum (FBS). Cells were incubated for 12 h for the migration assay and 24 h for the invasion assay at 37°C in an incubator at 5% CO_2_. After the indicate times, cultured cells that migrated through the membrane were fixed with methanol and stained with 0.5% crystal violet. Cell numbers were counted in five randomly chosen microscopic fields (100×) per membrane.

### Wound-Healing Assay

After transfection of K1 and TPC1 cells for 24 h, K1 and TPC1 cells were seeded in six−well plates until they reached 80% confluency. A 200-μl pipette tip was used to create scratches. Cells were washed with phosphate-buffered saline (PBS) and cultured in serum-free medium. The scratches were observed and photographed at 0 and 24 h after wounding in three randomly observed fields. The percentage of wound closure was measured using Image J software (NIH, Bethesda, MD, USA).

### Western Blotting

A Protein Extraction Kit (KeyGEN, Nanjing, China) was used to extract the proteins in PTC cells and tissues, and from adjacent non-cancerous thyroid tissues. Total proteins (40–50 μg) were subjected to sodium dodecyl sulfate polyacrylamide gel electrophoresis (SDS-PAGE). The separated proteins were then transferred electrophoretically onto 0.45-μm polyvinylidene difluoride membranes (Millipore, Billerica, MA, USA). The membranes were blocked using 5% skim milk, and then incubated with primary antibodies diluted with primary antibody dilution buffer, including those recognizing glyceraldehyde-3-phosphate dehydrogenase (GAPDH) (1:1,000 dilution, ZSGB-Bio, Beijing, China), TGM2 (1:2,000, ab109200, Abcam, Cambridge, MA, USA), p65 (1:2,000, ab32536, Abcam), and FN1 (1:1,000 dilution, 15613-1-AP Proteintech, Rosemont, IL, USA), at 4°C overnight. Subsequently, the antibodies bound to the membranes were reacted with secondary antibodies (1:1,000 dilution, ZSGB-Bio). The immunoreactive protein bands were detected using an enhanced chemiluminescence (ECL) kit from Thermo Fisher Scientific (Waltham, MA, USA).

### Immunohistochemistry (IHC)

Immunohistochemistry for the target molecules was performed on paraffin sections using a primary antibody recognizing TGM2 (1:200; ab2386, Abcam). The semi-quantitative Remmele scoring system was used to score the immunohistochemical staining.

### Immunofluorescence Staining

PTC cells at 1.5 × 10^5^ cells per well were transfected and cultured in six-well plates with cell slides. When the cells reached 70% confluence, they were subjected to fixation in 4% polyformaldehyde at room temperature for 15 min. After treatment with 0.3% Triton X-100 and blocking with goat serum for 30 min, the cells were incubated with anti-p65 antibodies (1:2,000, ab32536, Abcam) at 4° overnight. Finally, the cells were mounted under a fluorescence inverted microscope, observed, and photographed.

### Tumor Xenograft Model

All animal procedures were conducted in accordance with the guidelines of the China Medical University Institutional Animal Care and Use Committee. For the tumor metastasis assay, K1 cells (1 × 10^6^ K1) stably transfected with the LV-empty vector, LV-shRNA-NEAT1_2+pcDNA-TGM2, or LV-sh NEAT1_2, were injected into nude mice *via* the tail vein, separately. Seven weeks later, the mice were euthanized and their lungs were removed. The images and number of metastatic nodules in the lungs were captured and counted.

### Luciferase Assay

*NEAT1_2*-wild type *(NEAT1_2-*Wt) and *NEAT1_2*-mutation type (*NEAT1_2*-Mt) constructs containing the putative binding sites or mutated the putative binding sites, for miR-491 were amplified and ligated into pMIR-REPORT, a firefly luciferase expression vector (Obio Technology, Shanghai, China). Lipofectamine 2000 (Invitrogen, Carlsbad, CA, USA) was used to transfect HEK 293T cells with *NEAT1_2*-Wt and *NEAT1_2*-Mt, together with the Renilla luciferase expressing vector pRL-TK (Promega, Madison, WI, USA), which were then transfected with the miR-491 mimic or NC. Similarly, cells were cotransfected with the wild-type *TGM2* 3′ UTR (*TGM2*-3′ UTR-Wt) or the mutated *TGM2* 3′ UTR (*TGM2*-3′UTR-Mt) together with the miR-491 mimic or NC. All the co-transfected cells were also transfected with pRL-TK. We also constructed a pcDNA3.1-NEAT1_2 wild-type plasmid (pcDNA3.1-NEAT1_2 Wt), which contained miR-491 binding sites, and pcDNA3.1-NEAT1_2 mutated plasmid (pcDNA3.1-NEAT1_2 Mt), which contained mutated miR-491 binding sites, to further verify that TGM2 was regulated by *NEAT1_2 via NEAT1_2*’s interaction with miR-491. Next, the region of the *FN1* promoter containing p65 binding sites was cloned into the luciferase reporter vector pGL3-Basic (Promega). Cells were then subjected to cotransfection with the indicated reporter plasmids and pcDNA-p65, or with negative controls. Finally, the luciferase activities in the cells were assessed as mentioned above.

### Bioinformatic Analyses

Correlation analysis of the downstream factors of TGM2 was performed in gene expression profiles from The Cancer Genome Atlas (TCGA) database (http://cancergenome.nih.gov/) using the R software (version 3.4.2). PTC samples (n = 497) were divided into TGM2 high-expression and TGM2 low-expression groups according to the upper quartile value of TGM2 expression levels. The top 200 significantly upregulated genes in the TGM2 high-expression group were summarized using Microsoft Excel 2010 (**Supplementary Material 1**). Functional analysis was performed using Kyoto Encyclopedia of Genes and Genomes (KEGG) pathway enrichment analysis and KOBAS 2.0 software. Gene set enrichment analysis (GSEA) (https://www.broadinstitute.org/) was used to explore whether the identified gene sets displayed statistically significant differences between the two groups. The statistical significance was determined using the normalized enrichment score (NES) and false discovery rate (FDR). Four online prediction tools, RegRNA 2.0 (http://regrna2.mbc.nctu.edu.tw/index.html), miRDB (http://www.mirdb.org/), Targetcan (http://www.targetscan.org/vert_72/), and ENCORI (http://starbase.sysu.edu.cn/panCancer.php) were used to determine whether the miRNAs could not only bind to *NEAT1_2*, but also target *TGM2*. The putative binding sequences of p65 in the *FN1* promoter were obtained from JASPAR (http://jaspar.genereg.net/).

### Statistical Analysis

All statistical analyses were performed using SPSS 13.0 software (IBM Corp., Armonk, NY, USA). The differences in the relative expression levels of TGM2, miR-491, and *NEAT1_2*, in PTC tissues and adjacent non-cancerous tissues were assessed using a Wilcoxon signed-rank test. The relationships between TGM2 or *NEAT1_2* expression and the patients’ clinicopathological characteristics were assessed using a chi-squared test. Comparisons between two independent groups used a two-tailed independent sample t test, Statistical significance was indicated by p-values < 0.05.

## Results

### *NEAT1_2* Expression Was High and Could Regulate TGM2 in PTC

**Figure 1:**
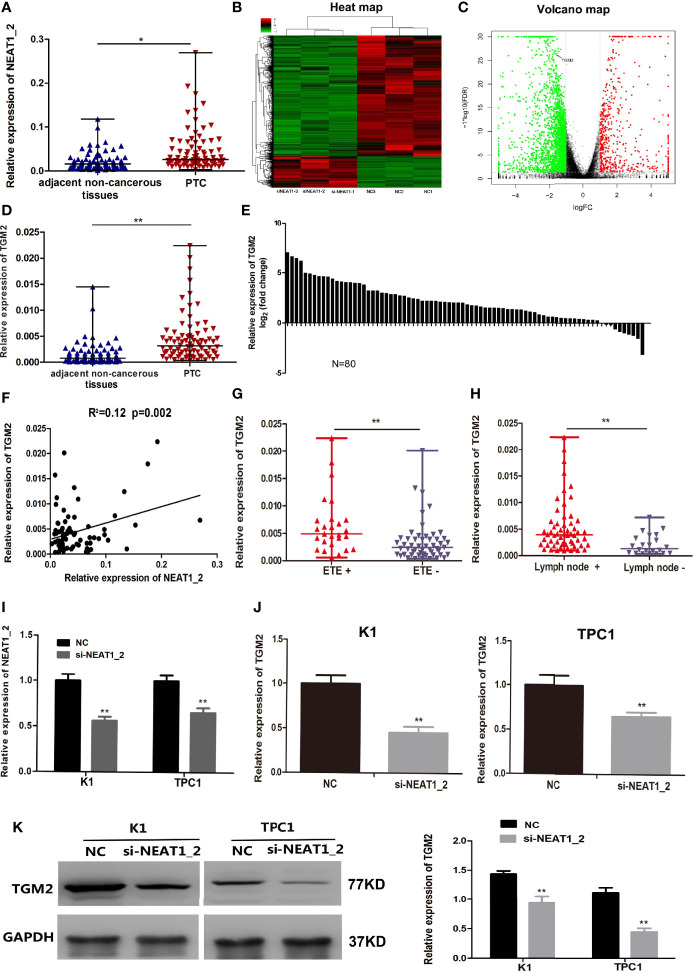
*NEAT1_2* expression was high and could regulate TGM2 in PTC. **(A)** The relative expression levels of *NEAT1_2* in 80 pairs of PTC tissues and adjacent non-cancerous tissues, as determined using qRT-PCR. The Wilcoxon signed-rank test was used to analyzed the differences between the two groups; data are presented as the median with a range. *P < 0.05. **(B)** Heat map of differentially expressed mRNAs (615 upregulated and 2364 downregulated) in K1 cells transfected with si-NEAT1_2 and NC. (Fold change ≥ 2, P < 0.05). **(C)** Volcano plots were constructed based on the sequencing results of the differentially expressed mRNAs between K1 cells transfected with si-NEAT1_2 and NC. The red points represent differentially upregulated genes, and green points represent downregulated genes (Fold change ≥ 2, P < 0.05). A red arrow indicates TGM2 in the plots. **(D)** The relative expression levels of *TGM2* in 80 pairs of PTC tissues and adjacent non-cancerous tissues, as determined using qRT-PCR. The Wilcoxon signed-rank test was used to analyzed the differences between the two groups; data are presented as the median with a range. **P < 0.01. **(E)** The fold change of TGM2 expression between PTC and corresponding adjacent non-cancerous tissues. **(F)** Pearson’s correlation analysis was performed to analyze the correlations between *NEAT1_2* and TGM2 expression in PTC tissues (R^2^ = 0.15, P < 0.05). **(G)** TGM2 expression in the extrathyroidal extension positive (n = 27) and extrathyroidal extension negative (n = 53) subgroups The Mann-Whitney U test was used to analyze the differences between the two groups; data are presented as the median with a range. **P < 0.01. **(H)** TGM2 expression in the lymph node metastasis positive (n = 58) and lymph node metastasis negative (n= 22) groups. The Mann-Whitney U test was used to analyze the differences between the two groups; data are presented as the median with a range. **P < 0.05. **(I)** qRT-PCR analysis of *NEAT1_2* interference efficiency after si-NEAT1_2 or NC transfection in PTC cells. **P < 0.01 *versus* NC. **(J)** The relative mRNA expression of *TGM2* was detected using qRT-PCR in PTC cells after transfection with si-NEAT1_2 or NC. Data are presented as the mean ± S.D., as analyzed using an independent samples t-test. **P < 0.01 *versus* NC. **(K)** Western blotting was applied to detect the protein level of TGM2 in PTC cells transfected with si-NEAT1_2 or NC. Data are presented as the mean ± S.D., as analyzed using an independent samples t-test. **P < 0.01 *versus* NC.

*NEAT1_2* expression was initially investigated using qRT-PCR in 80 PTC tissues and their adjacent non−cancerous tissues. Compared with that in adjacent non-cancerous tissues, *NEAT1_2* expression was markedly increased in PTC tissues (**Figure 1A**). Next, to investigate the downstream targets of *NEAT1_2*, we performed mRNA-seq in the *NEAT1_2* knockdown group and NC group in the K1 cell line. We found that after knockdown of *NEAT1_2*, 615 mRNAs were upregulated and 2364 mRNAs were downregulated. Among the downregulated mRNAs, *TGM2* was identified as a downstream target, which was downregulated by 66.67% in the *NEAT1_2* knockdown group compared with that in the NC group (p < 0.01) (**Figures 1B, C**). The regulatory relationship between *NEAT1_2* and TGM2 was further confirmed in PTC tissues and cell lines. Compared with that in the non-cancerous tissue group, TGM2 expression was significantly increased in the PTC group (**Figures 1D, E**). Spearman’ s correlation analysis indicated that a positive relationship existed between *NEAT1_2* and TGM2 (R^2^ = 0.12, p < 0.05) (**Figure 1F**). Exthyroidal extension and lymph node metastasis were positively associated with the large difference in relative TGM2 expression in PTC tissues (both p < 0.01) (**Figures 1G, H**). Upon knockdown of *NEAT1_2* expression in PTC cells, the expression of TGM2 mRNA or protein was significantly lower in the *NEAT1_2* knockdown group compared with that in the control group (**Figures 1I–K**). Thus, *NEAT1_2* might function by regulating the expression of TGM2.

### Upregulation of TGM2 Expression Promoted the Migration and Invasion in PTC Cells

**Figure 2:**
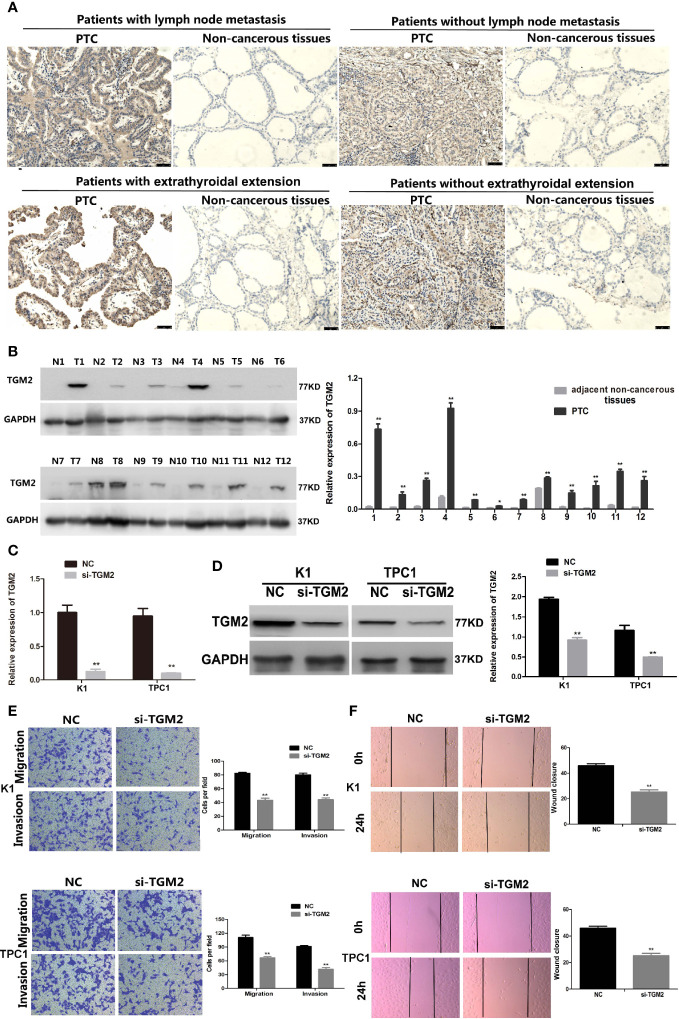
Upregulation of TGM2 expression promoted the migration and invasion of PTC cells. **(A)** Representative photographs from IHC analysis of TGM2 protein levels in normal and tumor samples, with or without lymph node metastasis and extrathyroidal extension. Scale bars: 50 μm. **(B)** Western blotting analysis of relative TGM2 protein levels in 12 pairs of PTC tissues (T) and corresponding adjacent non-cancerous tissues (N). *P < 0.05, **P < 0.01 *versus* adjacent non-cancerous tissues. **(C)** The relative mRNA expression of *TGM2* was detected using qRT-PCR in PTC cells after transfection with si-TGM2 or NC. Data are presented as the mean ± S.D., as analyzed using an independent samples t-test. **P < 0.01 *versus* NC. **(D)** Western blotting was applied to detect the protein level of TGM2 in PTC cells transfected with si-TGM2 or NC. Data are presented as the mean ± S.D., as analyzed using an independent samples t-test. **P < 0.01 *versus* NC. **(E)** Transwell assays were used to evaluate the migration and invasion in PTC cells after transfection with si-TGM2 or NC. Data are presented as the mean ± S.D., as analyzed using an samples t-test. **P < 0.01 *versus* NC. **(F)** A wound healing assay was applied to analyze the migration capacity in PTC cells after transfection with si-TGM2 or NC. All data are presented as the mean ± S.D.

Protein levels of TGM2 were detected *via* immunohistochemistry (IHC) and western blotting. To analysis whether TGM2 levels were associated with PTC clinicopathological features, IHC scoring was applied. As shown in **Table 1** and **Figure 2A**, significantly increased TGM2 protein levels were observed in PTC tissues, and positive expression of TGM2 was significantly related to lymph node metastasis (P = 0.003) and extrathyroidal extension (P = 0.035). PTC tissue overexpression of TGM2 was verified using western blotting using samples from 12 pairs of PTC and adjacent normal tissues (**Figure 2B**). To determine the functional roles of TGM2 in PTC, we further downregulated *TGM2* expression to investigate whether inhibition of TGM2 could affect PTC cell biological activity. The knockdown efficiency of si-TGM2 is shown in **Figures 2C, D**. Transwell assays demonstrated that after si-TGM2 transfection, PTC cell invasion and migration abilities were decreased compared with those of the NC group (**Figure 2E**). The effect of *TGM2* knockdown on migration was confirmed using a wound-healing assay (**Figure 2F**). Taken together, these results revealed that TGM2 was overexpressed in PTC and promoted the migration and invasion in PTC cells.

**Table 1:** Correlation between TGM2 expression and clinicopathological features in patients with papillary thyroid cancer (n = 92).

Characteristic	n	TGM2		P value
		+	−	
Age				
<55	69	59 (85.5%)	10 (14.5%)	0.204
≥55	23	17 (73.9%)	6 (26.1%)	
Gender				
male	36	31 (86.1%)	5 (13.9%)	0.477
female	56	45 (80.4%)	11 (19.6%)	
Tumor size				
<2cm	65	53 (81.5%)	12 (18.5%)	0.674
≥2cm	27	23 (85.2%)	4 (14.8%)	
Extrathyroidal invasion				
yes	45	41 (91.1%)	4 (8.9%)	0.035*
no	47	35 (74.5%)	12 (25.5%)	
Lymph node metastasis				
yes	59	54 (91.5%)	5 (8.5%)	0.003*
no	33	22 (66.7%)	11 (33.3%)	
Multicentricity				
Yes	49	39 (79.6%)	10 (20.4%)	0.415
No	43	37 (86.0%)	6 (14.0%)	
TNM stage				
I–II	58	45 (77.6%)	13 (22.4%)	0.097
III–IV	34	31 (91.2%)	3 (8.8%)	

*p < 0.05.

### The Overexpression of TGM2 Partially Impairs *NEAT1_2* Depletion-Mediated Migration and Invasion in PTC Cells or *In Vitro*

**Figure 3:**
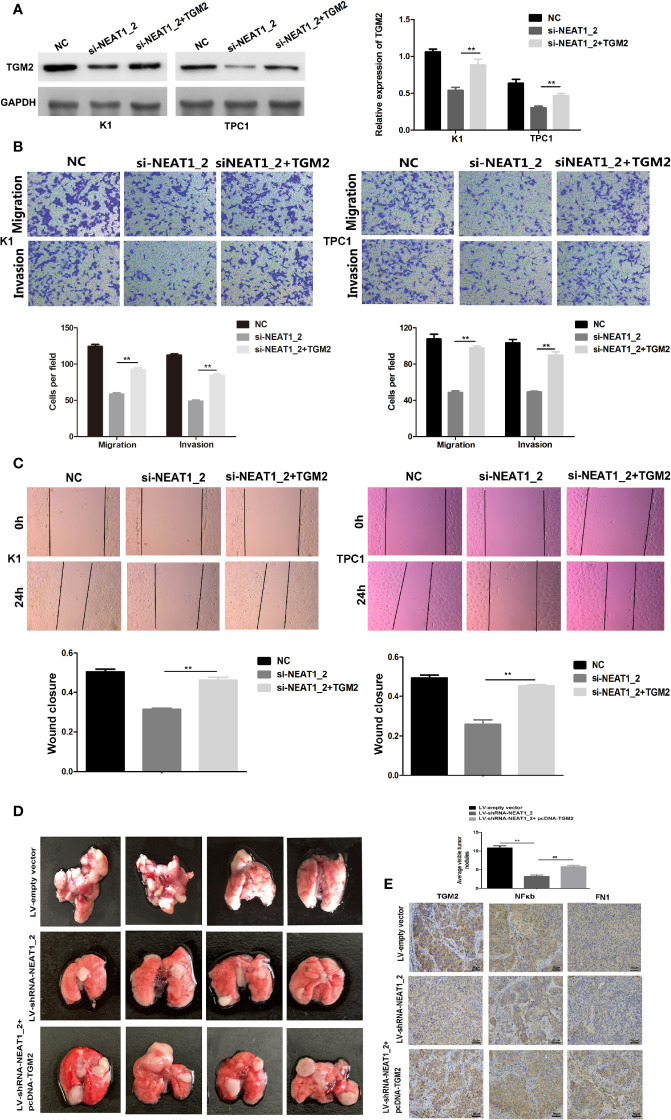
Overexpression of TGM2 partially impairs *NEAT1_2* depletion-mediated migration and invasion in PTC cells or *in vitro*. **(A)** Western blotting was used to analyze the level of TGM2 in PTC cells transfected with si-NEAT1_2 or co-transfected pCDNA3.1-TGM2 and si-NEAT1_2 or NC. Data are presented as the mean ± S.D., as analyzed using an independent samples t-test. **P < 0.01 *versus* NC. **(B)** Transwell assay analysis was used to evaluate the migration and invasion of PTC cells after transfection with si-TGM2 or after co-transfection pCDNA3.1-TGM2 and si-NEAT1_2 or NC. Data are presented as the mean ± S.D., as analyzed using an independent samples t-test. **P < 0.01 *versus* the si-NEAT1_2 group. **(C)** A wound healing assay was applied to analyze the migratory capacity of PTC cells after transfection with si-NEAT1_2 or co-transfected pCDNA3.1-TGM2 and si-NEAT1_2 or NC. **(D)** Lung metastasis nodules from model mice after intravenous injection of 1 × 10^6^ K1 cells of the LV-sh-NEAT1_2 group, the LV-empty vector group, and the LV-shRNA-NEAT1_2 + pcDNA-TGM2 group. Data are presented as the mean ± S.D., as analyzed using an independent samples t-test. **P < 0.01 *versus* LV-empty vector group, ^##^P < 0.01 *versus* LV-sh-NEAT1_2 group. **(E)** IHC staining to analyze FN1, NFκb, and TGM2 expression in the mouse lung tissue.

To investigate whether TGM2 mediated the invasion-inhibition effects of *NEAT1_2* knockdown in PTC cells, construct pCDNA3.1-TGM2 was cotransfected with si-NEAT1 into K1 or TPC1 cells. **Figure 3A** shows the western blotting results for TGM2 expression in the NC group, si-NEAT1_2 group, and the co-transfected si-NEAT1_2+TGM2 group. Compared with that in the si-NEAT1_2 group, the TGM2 levels increased significantly in the co−transfected group. Subsequently, functional experiments showed that the si-NEAT1_2-induced inhibition of migration and invasion was partly impaired by overexpression of TGM2 (**Figures 3B, C**). To further investigate TGM2’s effect PTC metastasis, a pulmonary metastasis assay was performed in nude mice by injecting LV-empty vector, LV-shRNA-NEAT1_2, and LV-shRNA-NEAT1_2+ pcDNA-TGM2 transfected cells into the mouse tail vein. Compared with those in the LV-empty vector group, the number of visible nodules in the LV-shRNA-NEAT1_2 group had decreased significantly (**Figure 3D**). The effects of co-transfected LV-shRNA-NEAT1_2+ pcDNA-TGM2 cells were similar to the effects of the LV-empty vector group in terms of invasion and metastasis. Moreover, the IHC staining to analyze FN1, NFκb, and TGM2 expression in the mouse lung tissue (**Figure 3E**). Thus, these results suggested strongly that in PTC cells or *in vivo*, *NEAT1_2* exerts an oncogenic function by targeting TGM2.

### *NEAT1_2* Regulates TGM2 *via* Downregulating miR-491

**Figure 4:**
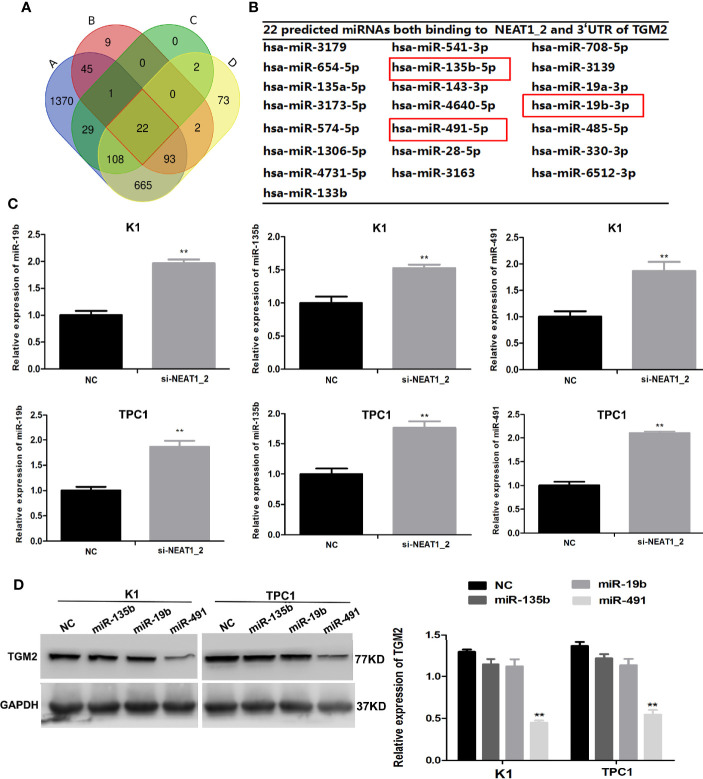
*NEAT1_2* regulates TGM2 *via* downregulating miR-491. **(A)** Identification of 22 common miRNAs that bind to both *NEAT1_2* and *TGM2* from four publicly available datasets (A: RegRNA 2.0, B: miRDB, C: Targetcan, and D: ENCORI). Different colored areas represent different datasets. **(B)** The 22 miRNAs that bind to both *NEAT1_2* and *TGM2* from bioinformatic prediction. **(C)** The relative expression levels of miR-19b, miR-135b, and miR-491 were detected using qRT-PCR in PTC cells transfected with si-NEAT1_2 or NC. Data are presented as the mean ± S.D., as analyzed using an independent samples t-test. **P < 0.01 *versus* NC. **(D)** Protein expression of TGM2 was detected using western blotting in PTC cells transfected with miR-19b mimic, miR-135b mimic, and miR-491 mimic or NC. Data are presented as the mean ± S.D., as analyzed using an independent samples t-test. **P < 0.01 *versus* NC.

Our results so far indicated that *NEAT1_2* promotes invasion and metastasis by targeting TGM2. To further understand the mechanism by which *NEAT1_2* inhibits the expression of TGM2 in PTC cells, we determined whether there were interactions between *NEAT1_2* and certain miRNAs, because it has been reported that lncRNAs may act as miRNA sponges. We used the target prediction and bioinformatic analysis tools RegRNA 2.0, miRDB, TargetScan, and ENCORI to identify potential miRNAs that could target *TGM2* and had *NEAT1_2* binding sites (**Figure 4A**). This analysis identified 22 miRNAs that were predicted to bind to both the 3′ UTR of *TGM2* and *NEAT1_2* (**Figure 4B**). Then, in K1 and TPC1 cells, we confirmed whether downregulation of *NEAT1_2* could upregulate the expression of the 22 predicted miRNAs, as assessed using qRT-PCR. The expression levels of miR-135b, miR−19b, and miR-491 were significantly lower in the NC group than in the *NEAT1_2* knockdown group (**Figure 4C**). Next, in K1 and TPC1 cells, we detected whether *TGM2* expression was downregulated by overexpressing miR-135b, miR-19b, and miR-491. *TGM2* expression was downregulated only by overexpression of miR-491 (**Figure 4D**). Thus, we believe that miR-491 regulates the TGM2 protein level in PTC cell lines.

### miR-491 Inhibited PTC Migration and Invasion in PTC and Could Directly Bind to *NEAT1_2* and the 3′ UTR of *TGM2*

**Figure 5:**
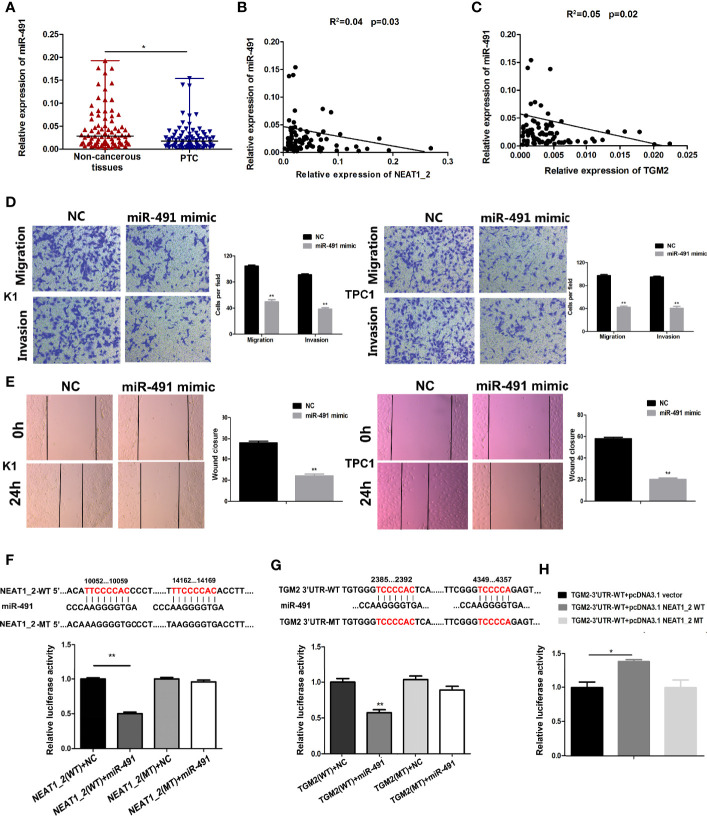
miR-491 inhibited PTC migration and invasion and could directly bind to *NEAT1_2* and the 3′ UTR of *TGM2*. **(A)** The relative expression levels of miR-491 in 80 pairs of PTC tissues and adjacent non-cancerous tissues, as determined using qRT-PCR. The Wilcoxon signed-rank test was used to analyze the differences between the two groups; data are presented as the median with a range. *P < 0.05. **(B)** Pearson’s correlation analysis was performed to analyze the correlations between *NEAT1_2* and TGM2 expression in PTC tissues (R^2^ = 0.056, P = 0.042). **(C)** Pearson’s correlation was performed to analyze the correlations between miR-491 and TGM2 expression in PTC tissues (R^2^ = 0.05, P = 0.029). **(D)** Transwell assays were used to evaluate the migration and invasion in PTC cells after transfection with miR-491 mimics or NC. Data are presented as the mean ± S.D., as analyzed using an independent samples t-test. **P < 0.01 *versus* NC. **(E)** A wound healing assay was applied to analyze the migration capacity in PTC cells after transfection with miR-491 mimics or NC. All data are presented as the mean ± S.D. **(F)** The predicted miR-491 binding sites in *NEAT1_2* (*NEAT1_2*-Wt) and the designed mutant sequence (*NEAT1_2*-Mt) are indicated. HEK 293T cells were transfected with *NEAT1_2*-Wt, *NEAT1_2*-Mt, and the indicated miRNAs, and then a luciferase reporter assay was conducted. Data are presented as the mean ± S.D., as analyzed using an independent samples t-test. **P < 0.01 *versus NEAT1_2*-Wt+NC. **(G)** The predicted miR-491 binding sites in the 3′-UTR region of *TGM2* (*TGM2*-3′UTR-Wt) and the designed mutant sequence (*TGM2*-3′UTR-Mt) are indicated. HEK 293T cells were transfected with *TGM2*-3′UTR-Wt or *TGM2*-3′UTR-Mt and the indicated miRNAs, and then a luciferase reporter assay was conducted. Data are presented as the mean ± S.D., as analyzed using an independent samples t-test. **P < 0.01 *versus TGM2*-3′UTR-Wt+NC. **(H)** *TGM2*-3′UTR-Wt was cotransfected with vectors pcDNA3.1, pcDNA3.1-NEAT1_2 Wt, and pcDNA3.1-NEAT1_2 Mt, respectively, and then a luciferase reporter assay was conducted. Data are presented as mean ± S.D., as analyzed using an independent samples t-test. **P < 0.01 *versus TGM2*-3′UTR-Wt+pcDNA3.1 vector.

To understand the role of miR-491 in PTC, we analyzed the expression of miR-491 in PTC tissues and non-cancerous tissues. qRT-PCR revealed that miR-491 was overexpressed in 80 paired non-cancerous tissues compared with its expression in PTC tissues (**Figure 5A**). In 80 PTC tissues, the expression of miR-491 correlated negatively and significantly with *NEAT1_2* or *TGM2*, according to the Pearson correlation test (**Figures 5B, C**). We further examined the effects of miR-491 on PTC progress. Transwell assays indicated that upregulation of miR-491 using its mimics decreased PTC migration and invasion activity in K1 and TPC1 cell lines (**Figure 5D**). In addition, a wound-healing assay suggested that overexpression of miR-491 in PTC cells led to a notable reduction in cell migration ability (**Figure 5E**). In addition, we confirmed that that miR-491 could bind to *NEAT1_2* and directly targeted *TGM2* using dual luciferase reporter assays. Luciferase activity was reduced significantly in cells co-transfected with *NEAT1_2*-Wt and the miR-491 mimic, while co-transfection with *NEAT1_2*-Mt and the miR-491 mimic did not change the luciferase activity (**Figure 5F**). Similarly, to ascertain whether *TGM2* is a direct target of miR-491, dual−luciferase reporter assays were performed. In HEK 293T cells, the luciferase activity from TGM2-3′UTR-Wt was reduced significantly after ectopic overexpression of miR-491; however, miR-491 overexpression did not affect the luciferase activity from TGM2-3′UTR-Mt (**Figure 5G**). Further dual luciferase reporter assays were performed to verify whether *NEAT1_2*’s interaction with miR-491 could regulate TGM2. The luciferase activity from TGM2-3′ UTR-Wt was increased significantly by pcDNA3.1-NEAT1_2 Wt; whereas pcDNA3.1-NEAT1_2 Mt did not alter the luciferase activity from TGM2-3′ UTR-Wt (**Figure 5H**). Thus, miR-491 inhibited PTC migration and invasion and could bind directly to the 3′ UTR of *TGM2* and *NEAT1_2*.

### Bioinformatic Analysis Showed That FN1 Is a Target of TGM2 in PTC

**Figure 6:**
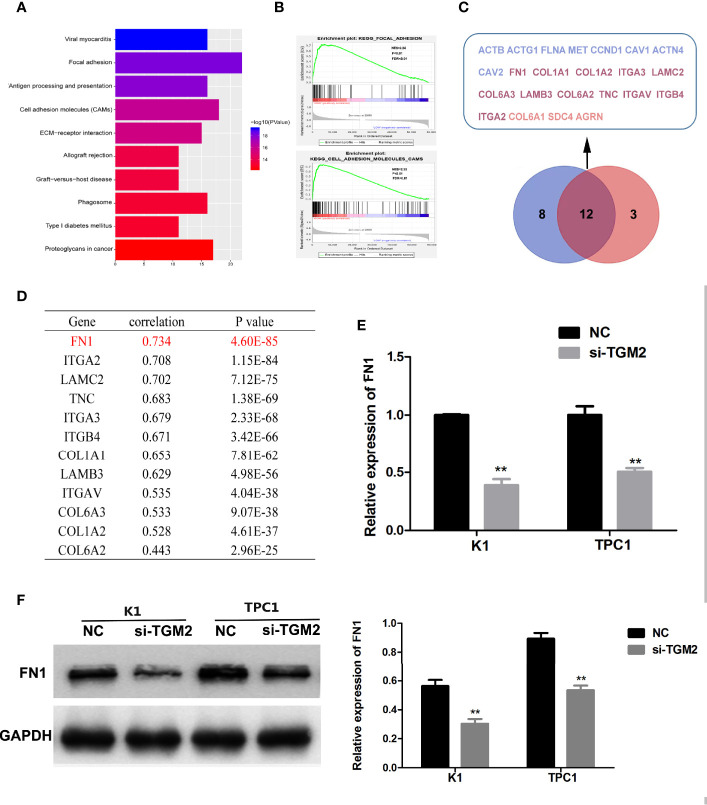
Bioinformatic analysis showing that FN1 is a target of TGM2 in PTC. **(A)** KEGG analysis of the top 200 differentially upregulated genes in TGM2 high-expression tumors. **(B)** GSEA highlighting the positive association of upregulated TGM2 expression levels with Focal adherence and Cell adhesion molecules. **(C)** Venn diagram analysis of the co-expressed genes in the set Focal adherence and Cell adhesion molecules. **(D)** Pearson’s correlation was performed to analyze the correlations between TGM2 and co-expressed genes in the set Focal adherence and Cell adhesion molecules. **(E)** The relative mRNA expression of *FN1* was detected using qRT-PCR in PTC cells after transfection with si-TGM2 or NC. Data are presented as the mean ± S.D., as analyzed using an independent samples t-test. **P < 0.01 *versus* NC. **(F)** Western blotting was applied to detect the protein level of FN1 in PTC cells transfected with si-TGM2 or NC. Data are presented as the mean ± S.D., as analyzed using an independent samples t-test. **P < 0.01 *versus* NC.

To further investigate the mechanism of TGM2’s involvement in PTC metastasis, we performed correlation analysis of TGM2 in gene expression profiles from the TCGA database. According to the upper quartile value of their TGM2 expression levels, PTC samples (n = 497) were classified as TGM2 high-expression and TGM2 low-expression. In the TGM2 high-expression group, Microsoft Excel 2010 was used to summarize the top 200 significantly upregulated genes (**Supplementary Material 1**). KEGG and GSEA using the TCGA thyroid cancer dataset was performed. As shown in **Figures 6A, B**, both KEGG and GSEA indicated that TGM2 was associated the function of Focal adherence and Cell adhesion molecules. By overlapping these two gene sets, 12 genes, which were identified with functions in both Focal adherence and Cell adhesion molecules, were screened using the VENNY tool (**Figure 6C**). Among these genes, the most relevant gene was *FN1* (encoding fibronectin) (r = 0.734, P < 0.0001), which is known to play an extremely important role in both focal adherence and cancer-related metastasis, and was chosen for further mechanistic study (**Figure 6D**). To confirm the regulatory relationship between TGM2 and FN1, we knocked down the expression of *TGM2* in PTC cell lines and found that the mRNA and protein levels of FN1 were dramatically downregulated (**Figures 6E, F**).

### TGM2 Transcriptionally Activates FN1 by Promoting p65 Nuclear Translocation in PTC Cell Lines

**Figure 7:**
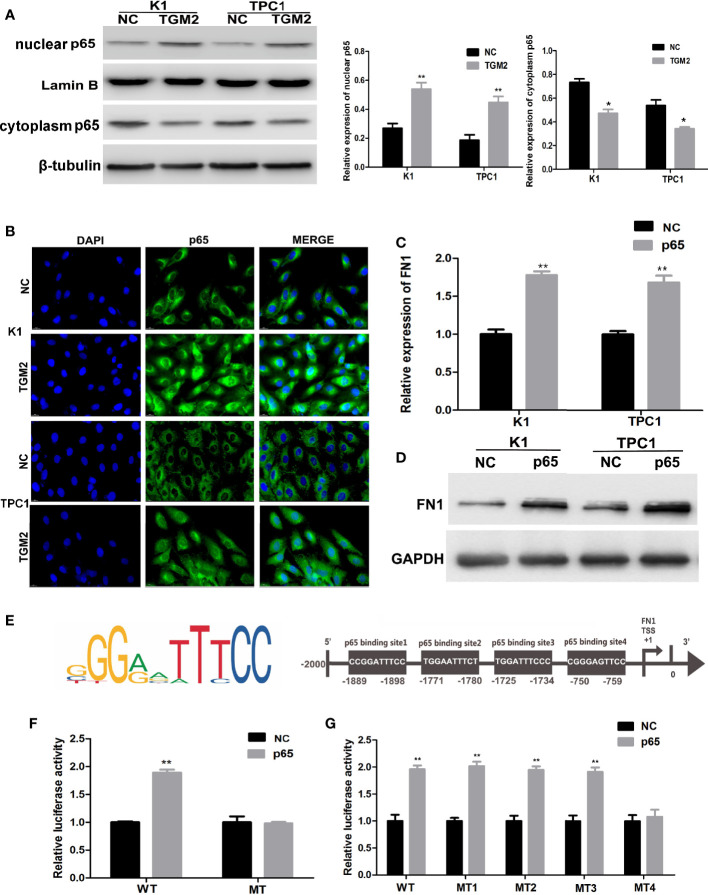
TGM2 transcriptionally activates FN1 by promoting p65 nuclear translocation in PTC cell lines. **(A)** Overexpression of TGM2 activates p65 nuclear translocation in PTC cells, as measured using western blot assays. Data are presented as the mean ± S.D., as analyzed using an independent samples t-test. *P < 0.05; **P < 0.01 *versus* NC. **(B)** Overexpression of TGM2 activates p65 nuclear translocation in PTC, as measured using immunofluorescence staining. **(C)** qRT-PCR was applied to detect the protein level of FN1 in PTC cells transfected with p65 or NC. Data are presented as the mean ± S.D., as analyzed using an independent samples t-test. **P < 0.01 *versus* NC. **(D)** Western blotting was applied to detect the protein level of FN1 in PTC cells transfected with p65 or NC. Data are presented as the mean ± S.D., as analyzed using an independent samples t-test. **P < 0.01 *versus* NC. **(E)** Prediction of the p65 binding site in the *FN1* promoter region using JASPAR (http://jaspar.genereg.net/). **(F)** The effect of overexpression of p65 on luciferase activity of a vector containing the *FN1* wild-type promoter or mutated promoter. Data are presented as the mean ± S.D., as analyzed using an independent samples t-test. **P < 0.01 *versus* NC. **(G)** The effect of p65 overexpression on the luciferase activity of a vector containing the *FN1* wild−type promoter or mutation site 1, mutation site 2, mutation site 3, or mutation site 4. Data are presented as the mean ± S.D., as analyzed using an independent samples t-test. **P < 0.01 *versus* NC.

TGM2 could regulate FN1 mRNA and protein expression; therefore, TGM2 might regulate FN1 at the transcription level. TGM2 is not a transcription factor. However, studies have reported that TGM2 could trigger cancer invasion by regulating master transcription factors (TFs) or promoting TF nuclear translocation, such as for C/EBPβ, STAT3, and p65 (19). Interestingly, we found that the upregulation of TGM2 could significantly promote the levels of p65 in the nucleus and decreased in cytoplasm (**Figure 7A**). Then, using a cell immunofluorescence assay, we found similar results: The p65 fluorescence intensity in the nucleus increased significantly in the TGM2 overexpression group compared with its level in the control group (**Figure 7B**). P65 overexpression resulted in significant upregulation of FN1 mRNA and protein levels (**Figures 7C, D**). To further explore the regulatory mechanism of FN1 in PTC, we matched the promoter sequences of *FN1* with potential transcription factors using JASPAR and found four potential binding sites for p65 in the *FN1* promoter (**Figure 7E**). Moreover, we performed a luciferase assay to further investigate the specific binding sites between p65 and the *FN1* promoter. As shown in **Figure 7F**, luciferase activity was elevated in wild-type group, but failed to affect that from mutation group, indicating that p65 could indeed bind to the promoter of *FN1*. To further detect the specific binding sites of p65 in *FN1*, we constructed a luciferase reporter vector containing the *FN1* promoter or containing mutant site 1, site 2, site 3, or site 4. The results showed that the luciferase activity resulting from p65 overexpression was abolished only when site 4 was mutated (**Figure 7G**). Therefore, site 4 was responsible for the transcription activation of p65 on *FN1*. All these results indicated that TGM2 transcriptional activated *FN1* by promoting p65 nuclear translocation in PTC cell lines.

## Discussion

The various forms of PTC are characterized as differentiated cancers, and PTCs alone account for more than 80% of all thyroid tissue malignancies (2). According to American Joint Committee on Cancer 8th stage for differentiated thyroid cancer, local invasion and distant metastasis are critical factors affecting the prognosis of patients with thyroid cancer (5). However, so far, there are no effective therapeutic targets and biomarkers for thyroid cancer with invasion and metastasis. Therefore, there is an urgent need to determine the molecular mechanism of PTC, which will allow the development of new and effective therapeutic approaches.

Over the past decades, aberrant expression of lncRNAs in cancers has been widely observed and has been reported to correlate with tumorigenesis. Our previous microarray study reported a genome-wide analysis on the expression profile of lncRNAs and identified 3499 lncRNAs that were differentially expressed (fold change ≥ 2.0, P < 0.05) in five paired PTC tissues and non-cancerous tissues (20). Among them, we found that *NEAT1_2*, a transcript of *NEAT1*, was significantly overexpressed in PTC tissues compared with adjacent non-cancerous tissues (fold change = 4.65, p < 0.05) (20). Recent studies indicated that *NEAT1* is upregulated and plays a functional role in tumorigenesis. For example, *NEAT1* can promote prostate cancer by regulating ACSL4 *via* sponging miR-34a-5p and miR-204-5p, and promotes the growth of gastric cancer cells by regulating the miR-497-5p/PIK3R1 axis (9, 21). Moreover, *NEAT1* expression is a novel prognostic and diagnostic biomarker in gastric cancer, colorectal cancer, esophageal squamous cell carcinoma, and prostate cancer (22). Our previous study demonstrated that knockdown of *NEAT1_2* induced apoptosis and inhibited the migration and invasion of PTC cells by regulating ATAD2 (12). However, rescue experiments indicated that ATAD2 was not the only downstream target of *NEAT1_2* (12). This means that there must be other downstream targets that mediate the invasion and metastasis of PTC. Thus, identifying further target genes of *NEAT1_2* in PTC invasion and metastasis was the main focus of this study.

Through mRNA sequencing of transfected si-NEAT1_2 K1 cells and NC K1 cell, we identified that *TGM2* was downregulated by 66.67% after NEAT1_2 knockdown. In 80-paired PTC tissues and non-cancerous tissues, the expression of *TGM2* mRNA correlated positively with that of *NEAT1_2*. Then, knockdown of *NEAT1_2* in two PTC cell lines using si-NEAT1_2 downregulated TGM2 mRNA and protein expression, suggesting that *NEAT1_2* could regulate the expression of TGM2 in PTC.

TGM2 is a complex and widely present member of the transglutaminase family, responsible for the calcium-dependent posttranslational modification of proteins, in which stable ϵ-(γ-glutamyl) lysine isopeptide linkages are introduced or polyamines are incorporated at certain peptide-bound glutamine residues (23). Recent studies have implicated TGM2 in various biological processes, including cell differentiation, extracellular matrix (ECM) stabilization, and cell migration (24). For example, TGM2 could regulate angiogenesis and apoptosis *via* Wnt/β-catenin pathway in colorectal cancer and TGM2 inhibition reversed mesenchymal transdifferentiation by regulating C/EBPβ signaling in glioma stem cells (25–27). However, whether TGM2 functions in PTC is unknown. The results of the present study demonstrated that PTC tissues overexpressed TGM2 compared with adjacent non-cancerous tissues. In addition, overexpression of TGM2 was associated with lymph node metastasis and extrathyroidal extension in PTC tissues. *TGM2* knockdown significantly affected PTC cell migration and invasion, suggesting an oncogenic role for TGM2 in PTC. A “rescue” strategy comprising cotransfecting pCDNA3.1-TGM2 and si-NEAT1_2 not only increased TGM2 protein expression, but also rescued the inhibition of migration, invasion, and metastasis in PTC cell lines or *in vitro*. Taken together, these results suggested that TGM2 acts as an oncogene in PTC invasion or metastasis, under the regulation of *NEAT1_2*.

LncRNAs can modulate miRNA functions by acting as ceRNAs (also known as molecular sponges) (28). In the development of different cancers, *NEAT1* has been observed to function as a ceRNA (9–11). This led us to speculate that *NEAT1_2* regulates TGM2 expression in PTC by acting as a ceRNA to sponge miRNAs. Bioinformatic analyses identified 22 miRNAs with binding sites for *NEAT1_2* and for the 3′ UTR of *TGM2*. qRT-PCR and western blotting showed that only miR-491 could reduce *TGM2* expression in PTC cells. Hence, we speculated that miR-491 could bind both the 3′ UTR of *TGM2* and *NEAT1_2*.

The gene encoding miR-491 resides in the 9p21.3 region, and recent studies have shown that miR-491 functions as an important tumor suppressor (29). miR-491 affects apoptosis and proliferation in pancreatic carcinoma, ovarian carcinoma, and breast cancer; and reduces the invasive behavior of human hepatocellular carcinoma, breast cancer, and oral squamous cancer (30–35). However, whether miR-491 functions in PTC is not clear. Consistently, we found that miR-491 was overexpressed in 80 paired non-cancerous tissues compared with that in PTC tissues. The expression of miR-491 was significantly inversely correlated with *NEAT1_2* and *TGM2* expression. Next, PTC cell invasion and migration were inhibited significantly by miR-491 overexpression. Dual luciferase reporter assays suggested that miR-491 is the specific miRNA that binds to both the 3′ UTR of *TGM2* and *NEAT1_2*. In addition, *NEAT1_2* sponges miR-491 to regulate TGM2 expression, revealing the mechanism by which *NEAT1_2* modulates TGM2 expression to promote migration or invasion of PTC.

KEGG and GSEA analysis of the top 200 significantly upregulated genes in the TGM2 high-expression group from the TCGA database found that high expression of TGM2 correlated significantly with Focal adherence and Cell adhesion molecules, which are significantly related to cancer invasion and metastasis (36). Among the genes that were highly positive correlated with TGM2 was *FN1*, which is involved in both Focal adherence and Cell adhesion molecules. FN1, a glycoprotein, has major roles in cell growth, differentiation, migration, and adhesion, and is vital for embryonic development and wound-healing (37). Degradation or alteration of FN1 expression has been associated with cancer progression, such as in squamous cell carcinoma, nasopharyngeal carcinoma, ovarian cancer, and renal cancer (37–39). Moreover, studies have reported that FN1 is overexpressed and associated with lymph node metastasis in PTC (40, 41). In the present study, we found that TGM2 could regulate the mRNA and protein of levels of FN1, indicating that TGM2 might regulate FN1 at the transcription level. Although TGM2 is not a TF, it can trigger cancer progression by regulating master TFs or promoting TF nuclear translocation. For example, TGM2 could form complexes with NFκb components and promote p65 nuclear translocation (15, 42). P65 could further activate IL−6 in mantle cell lymphoma cells, or hypoxia inducible factor (HIF), vascular endothelial growth factor (VEGF) and EMT-associated proteins in breast cancer (15, 42). Bioinformatic prediction identified four binding sites between p65 and the promoter of *FN1*. Thus, we speculated that TGM2 might regulate *FN1* transcription by promoting p65 nuclear translocation. Upregulation of TGM2 significantly promoted the p65 level in the nuclei of PTC cell lines, and overexpression of p65 significantly upregulated the mRNA and protein levels of FN1. Luciferase assays using wild-type and mutant FN1 promoters confirmed that p65 binds to the FN1 promoter *via* binding site 4 among four putative binding sites. All these results suggested that TGM2 promoted the nuclear translocation of p65, where p65 transcriptional activates *FN1*, finally promoting invasion and metastasis of PTC.

## Conclusions

In conclusion, our study identified that the lncRNA *NEAT1_2* might act as a ceRNA to sponge miR-491, thus regulating TGM2 expression. TGM2 could transcriptionally activate *FN1 via* the binding of p65 to the *FN1* promoter, thereby promoting the invasion and metastasis of PTC. These data regarding the multiple mechanisms of *NEAT1_2* in PTC increase our understanding of the detailed molecular mechanisms associated with PTC progression.

## Data Availability Statement

The datasets presented in this study can be found in online repositories. The names of the repository/repositories and accession number(s) can be found below: https://www.ncbi.nlm.nih.gov/geo/query/acc.cgi?acc=GSE159905.

## Ethics Statement

The studies involving human participants were reviewed and approved by the Ethics Committee of the First Affiliated Hospital of China Medical University. The patients/participants provided their written informed consent to participate in this study. All animal studies were conducted in accordance with the principles and procedures outlined in the guidelines of the Institutional Animal Care and Use Committee (IACUC) of China Medical University (IACUC approval number: TZ2019137).

## Author Contributions

Conceived and designed the experiments: WS, HZ, WD, ZW, LH, TZ, and YQ. Performed the experiments: WS and YQ. Analyzed the data: WS and HZ. Wrote the paper: WS. All authors contributed to the article and approved the submitted version.

## Funding

This work was supported by the National Natural Science Foundation of China (grant number 81902726), the Project funded by China Postdoctoral Science Foundation (grant number 2018M641739), and Natural Science Foundation of Liaoning Province (grant number 20180530090).

## Conflict of Interest

The authors declare that the research was conducted in the absence of any commercial or financial relationships that could be construed as a potential conflict of interest.

## Supplementary Material

Supplement Material 1 The top 200 significantly upregulated genes in the TGM2 high-expression group.
